# Acute whole-body vibration therapy enhances cognitive speed without altering heart rate variability in healthy young adults

**DOI:** 10.3934/Neuroscience.2025028

**Published:** 2025-11-13

**Authors:** Zaw Myo Hein, Nisha Shantakumari, Yazan Ayman Khaoli, Che Mohd Nasril Che Mohd Nassir

**Affiliations:** 1 Department of Basic Medical Sciences, College of Medicine, Ajman University, P.O. BOX 346, Ajman, United Arab Emirates; 2 Center of Medical and Bio-Allied Health Sciences Research (CMBHSR), Ajman University, P.O. BOX 346, Ajman, United Arab Emirates; 3 Department of Anatomy and Physiology, Faculty of Medicine, Universiti Sultan Zainal Abidin, 20400 Kuala Terengganu, Terengganu, Malaysia

**Keywords:** whole body vibration, therapy, cognitive function, heart rate variability, Stroop Test, executive function

## Abstract

Whole-body vibration therapy (WBVT) is increasingly recognized as an alternative exercise modality with established benefits for musculoskeletal and cardiovascular health. Its effects on cognitive performance and autonomic regulation in healthy young adults, however, remain unclear. This within-subject study investigated whether acute WBVT influences executive function and heart rate variability (HRV). Thirty-six healthy volunteers (aged 18–25 years) completed two testing sessions separated by 7 days: a baseline (no vibration) session and a single 10-min WBVT session performed in a standing posture. Cognitive performance was assessed immediately after each session using a modified Stroop test, and electrocardiographic recordings were analyzed for HRV indices, including stress index, low-frequency (LF) and high-frequency (HF) power, LF/HF ratio, and mean heart rate. Compared with baseline, WBVT was associated with faster mean reaction times for congruent and incongruent Stroop stimuli (all p < 0.001) without changes in response accuracy. The stress index increased during the Stroop task relative to baseline (p = 0.052) and returned toward baseline following WBVT, whereas LF, HF, the LF/HF ratio, and total power showed no statistically significant changes. In this cohort, acute WBVT was associated with improved processing speed without measurable alterations in standard HRV metrics. These preliminary findings suggest that WBVT may transiently facilitate attentional processing in healthy young adults, but controlled trials with sham conditions and mechanistic measures are needed to confirm and contextualize these effects.

## 1. Introduction

Whole-body vibration therapy (WBVT) is an exercise modality in which mechanical oscillations are transmitted through the body via a vibrating platform. It has gained attention both as an alternative and as an adjunct to conventional exercise programs, with reported benefits for musculoskeletal health, cardiovascular fitness, and metabolic regulation [1]–[4]. Moreover, WBVT is increasingly conceptualized as a form of passive exercise, whereby externally applied oscillations elicit physiological responses without the voluntary muscular contractions typically required in active exercise [5]. By applying vibrations in the range of approximately 15–60 Hz, WBVT produces mild physiological stress that can trigger adaptive responses across neuromuscular, endocrine, and circulatory systems [2].

Beyond its physical effects, an emerging line of research has begun to examine how WBVT influences the central nervous system. Somatosensory stimulation from vibration has been reported to increase cortical excitability in motor and prefrontal regions, potentially modulating processes such as attention, working memory, and executive control [6]–[8]. In animal models, mechanical vibration appears to promote neuroplasticity through increased hippocampal neurogenesis, altered neurotransmitter release, and upregulation of brain-derived neurotrophic factor (BDNF) [9]–[11]. Theoretically, these cognitive effects can be framed within the Yerkes–Dodson law, which proposes that moderate physiological arousal may optimize performance by enhancing attentional resources and processing efficiency [12],[13]. In this context, WBVT may act as a controlled arousal stimulus that transiently improves selective attention and cognitive control without triggering excessive stress. Neuroimaging studies also suggest that vibrotactile and proprioceptive somatosensory input, particularly from mechanoreceptors in the plantar surface and muscle spindles, can recruit distributed cortical networks, including the anterior cingulate and dorsolateral prefrontal cortices, critical for executive function [14],[15].

However, human studies investigating the acute cognitive effects of WBVT are limited, and findings remain inconsistent. For instance, Regterschot et al. [16] reported that a single WBVT session acutely enhanced executive performance in older adults, while Tsai [17] demonstrated that chronic WBVT over several weeks improved working memory in community-dwelling adults. Some reports have shown improvements in reaction time (RT) and cognitive flexibility, particularly in older adults or individuals with cognitive impairment [18],[19]. In young healthy adults, data are sparse; Yoon et al. [20] found that low-frequency WBVT mitigated cognitive fatigue and accelerated RT, whereas other work suggests variable or no effects, likely due to differences in vibration parameters, task paradigms, and participant characteristics [21]. Studies vary widely in terms of vibration parameters (frequency, amplitude, and session duration), stimulation paradigms (side-alternating vs. vertical), and cognitive assessments (e.g., Stroop test, n-back, trail-making), which complicates direct comparison. This highlights the need to systematically test both acute and chronic WBVT protocols in well-defined cognitive domains.

The association between autonomic regulation and cognitive performance is well described in the neurovisceral integration model, which posits that prefrontal–subcortical networks regulate both executive control and autonomic flexibility via vagal pathways [22]–[25]. Empirical evidence demonstrates that higher heart rate variability (HRV) is linked with superior attentional control, working memory, and inhibitory processes [26]–[28]. This bidirectional brain–heart interaction provides a framework to test whether WBVT, like active exercise, can modulate cognitive performance through autonomic mechanisms [29],[30]. However, whether such effects generalize to passive exercise modalities such as WBVT remains largely unexplored.

Taken together, while active physical exercise is consistently shown to enhance HRV and cognition, it remains unclear whether WBVT, as a form of passive exercise, elicits comparable effects. Moreover, few studies have jointly examined cognitive and autonomic outcomes, leaving a critical gap in understanding the psychophysiological mechanisms underlying WBVT. To address these gaps, we investigated the acute effects of WBVT on cognitive performance and autonomic function in healthy young adults. We focused on selective attention and response inhibition, key components of executive function sensitive to arousal state. The modified Stroop test was selected as a robust, validated measure of interference control and attentional processing [31],[32]. HRV indices and stress indices were assessed to capture autonomic responses. We hypothesized that a single WBVT session would be associated with faster processing speed on the Stroop task, without producing maladaptive changes in standard HRV metrics, thereby providing preliminary evidence for WBVT as a safe, non-invasive approach to transiently facilitate cognitive performance.

## 2. Materials and methods

### 2.1. Study design

The study adopted a within-subject, pretest–posttest crossover design, consistent with the taxonomy proposed by Pontifex et al. [33]. Each participant served as their own control and completed two experimental sessions: a baseline (no-vibration) session and a WBVT session, conducted in counterbalanced order and separated by a 7-day interval. Pre- and post-assessments of cognitive and autonomic function were performed immediately before and after each session to capture acute within-session effects, while the 7-day gap ensured independent baseline states for comparison. Participants were recruited via flyers posted across campus, classroom announcements, and word of mouth.

**Figure 1.**
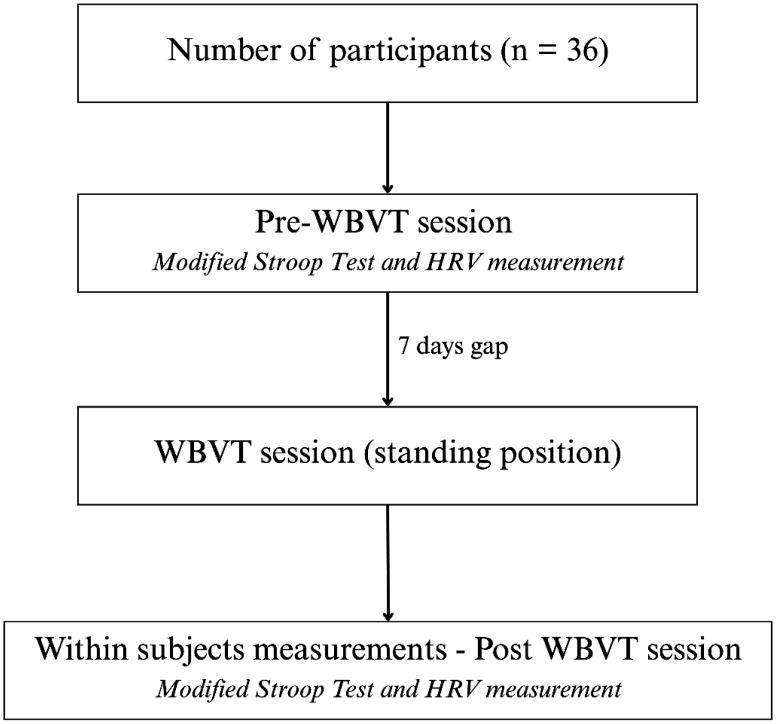
Flow diagram of participant progression through the study protocol. Participants (n = 36) first underwent a pre-intervention session that included the modified Stroop test and heart rate variability (HRV) measurement. After a 7-day interval, participants received WBVT in a standing position, followed by a post-intervention session in which the same assessments were repeated.

Participants (n = 36) were instructed to refrain from exercise, smoking, and caffeine consumption for at least two hours before each session to minimize confounding effects on autonomic and cognitive outcomes. As illustrated in Figure 1, all participants initially completed a pre-WBVT assessment, which included the modified Stroop test and HRV measurements. After a 7-day interval, they underwent a single WBVT session in a standing position, followed by a post-WBVT assessment involving the same cognitive and physiological tests. Although Figure 1 shows a single WBVT session, the broader protocol included alternating WBV and resting conditions in a standing posture. Randomization was performed using the randomization module in SPSS v26 (IBM Corp., Armonk, NY) with gender-stratified allocation to ensure balanced order of conditions, conducted independently with a 1:1 allocation ratio to control for potential order effects.

The 7-day interval between the pre-WBVT and WBVT sessions was intentionally implemented to ensure methodological rigor and minimize potential confounding effects [34]. This interval helps reduce short-term carryover from the initial testing session, such as mental fatigue, cognitive priming, arousal-related facilitation, or residual autonomic activation that may persist for several hours after testing [29]. A one-week spacing also provides sufficient time for participants to return to their physiological and cognitive baseline, which is critical in within-subject designs evaluating acute interventions. Moreover, heart rate variability indices have demonstrated high test–retest reliability across one week, supporting the validity of this intersession interval for autonomic assessments [35]. The 7-day separation further reduces the likelihood of adaptation or habituation to both the modified Stroop test and WBVT stimuli, preserving the sensitivity of outcome measures [36]. In addition, this interval helps control for daily fluctuations in HRV and cognitive performance arising from lifestyle variables such as sleep, stress, or caffeine intake [37]. Overall, the 7-day gap was chosen to ensure that any observed post-WBVT changes in HRV or cognitive performance could be attributed more confidently to the acute physiological effects of WBVT rather than to transient carryover or practice effects.

### 2.2. Ethical approval

The study was approved by the Human Research Ethics Committee at Ajman University (approval number M-F-H-4-Dec) and conducted in accordance with the Declaration of Helsinki. Written informed consent was obtained from all participants before enrollment.

### 2.3. Sample size calculations and study participants

A priori sample size estimation was performed using G*Power (Version 3.1). Based on prior WBVT studies reporting medium effect sizes (Cohen's d ≈ 0.5) for changes in reaction time and autonomic measures [16],[17], we calculated the number of participants required to detect a within-subject mean difference with 80% statistical power at an alpha level of 0.05 (two-tailed). The analysis indicated that at least 34 participants were needed to detect a medium effect (*f* = 0.25) in repeated-measures comparisons across two sessions. To account for potential data loss due to artifacts or non-compliance, we recruited 36 participants. This sample size provided sufficient power to detect clinically meaningful within-subject differences in both cognitive and autonomic outcomes while maintaining feasibility for a controlled laboratory study.

Thirty-six healthy young adults were recruited from Ajman University. Inclusion criteria were age 18–25 years, right-handed, and normal or corrected-to-normal vision. Exclusion criteria included self-reported psychiatric, neurological, cardiovascular, endocrinological, or orthopedic disorders, use of psychoactive medications, color vision deficiency, and substance use. Participants completed a standardized health-screening questionnaire adapted from the Physical Activity Readiness Questionnaire (PAR-Q) [38],[39], supplemented with additional custom items to capture relevant medical history (e.g., prior neurological or cardiovascular disorders, current medication use, recent injuries, and substance intake). Individuals reporting a positive response to PAR-Q items or custom exclusion criteria were not enrolled.

Anthropometric measures (height, weight) were obtained using a calibrated stadiometer (Seca 213, Seca GmbH & Co. KG, Hamburg, Germany) and digital scale (Omron HN-289, Omron Healthcare Co., Kyoto, Japan). BMI was calculated as body mass (kg) divided by height squared (m²). Health status was assessed via a self-reported medical questionnaire covering cardiovascular, neurological, orthopedic, and endocrinological conditions. Physical activity levels were recorded using the short form of the International Physical Activity Questionnaire (IPAQ-SF), which has established reliability and validity in young adults [40].

### 2.4. WBVT intervention

WBVT was delivered using the WBV Bluefin Fitness 3D Vibration Plate (Bluefin Trading International, Denmark). Actual vibration characteristics were quantified using a triaxial accelerometer (Actigraph GT9X Link) positioned at the midfoot while a participant stood on the platform. Frequency and peak-to-peak displacement were measured in the x, y, and z axes (see Table 1). Acceleration was calculated from displacement and frequency, assuming sinusoidal motion.

**Table 1.** Results of the actual frequency and peak-to-peak displacement.

**Parameters**	**Standing**
Frequency (Hz)	x: 27.1
	y: 27.1
	z: 27.1
Peak-to-peak displacement (mm)	x: 0.57
	y: 0.05
	z: 1.39

Note: Frequency and peak-to-peak displacement were measured at the medial side of the midfoot, while the vibrating platform was loaded with a participant for each posture.

**Figure 2.**
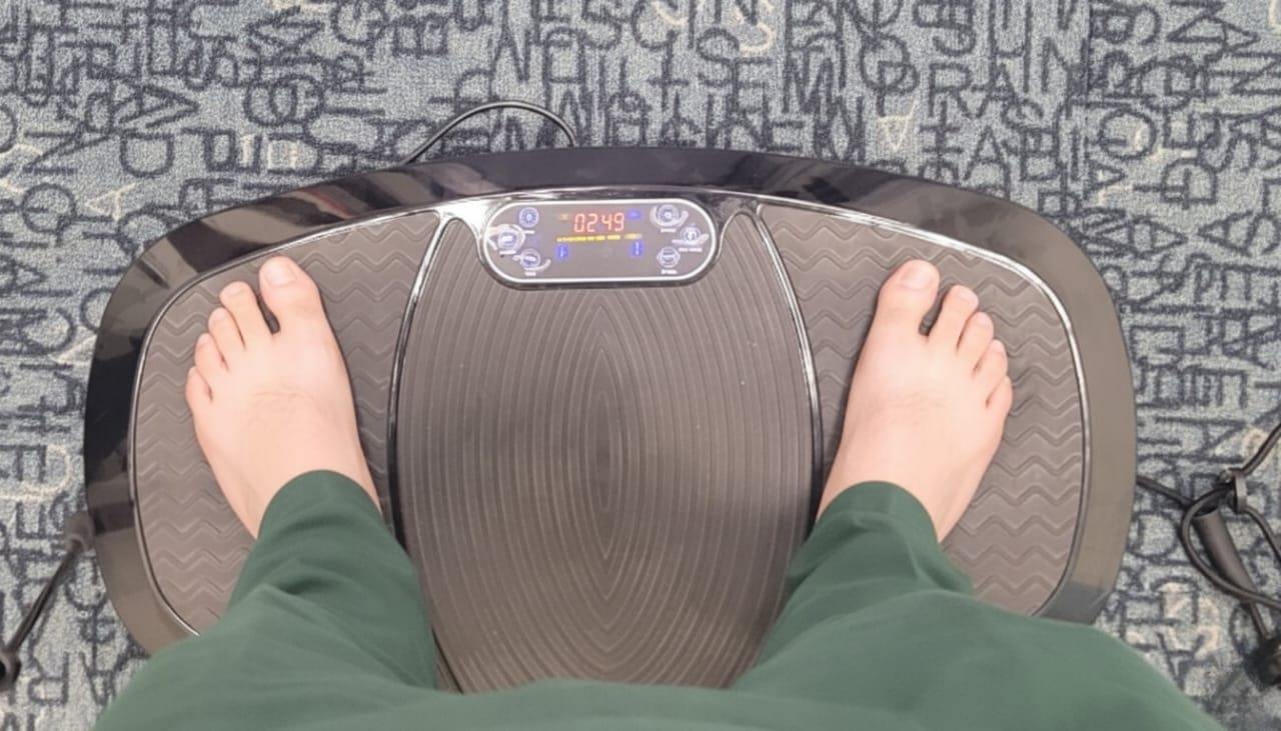
Feet positioning on the whole-body vibration (WBV) Bluefin Fitness 3D Vibration Plate in standing postures.

**Figure 3.**
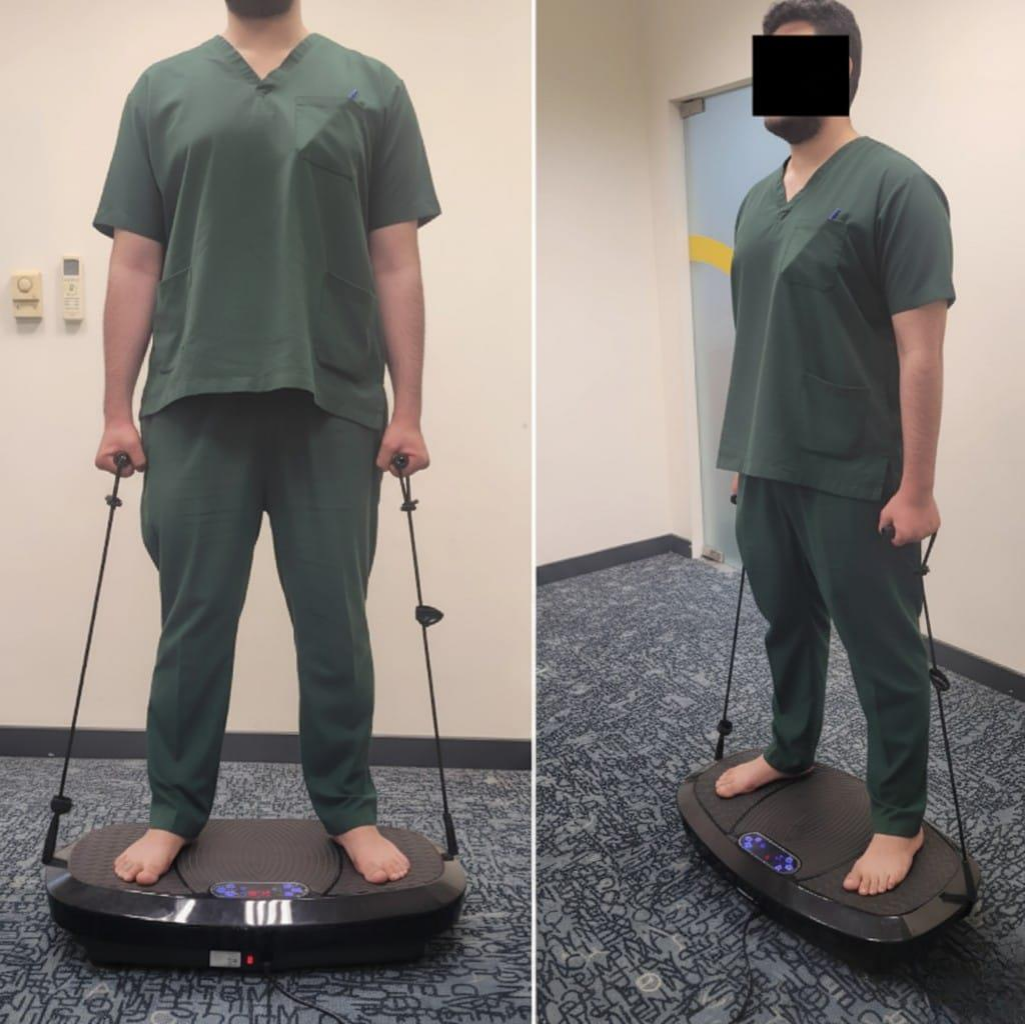
Standing posture is used during the whole-body vibration therapy (WBVT) sessions. Participants stood on the vibration platform in a static light position while holding the handrails for stability. This standardized posture was maintained throughout each WBVT bout to ensure consistent exposure and minimize postural variability.

All participants experienced a 10-min session of WBVT. WBVT equipment features a vibration platform that oscillates around an axis in alternating up-and-down motions (Figure 2). Participants stood barefoot on the platform in an upright position while keeping their feet on the outer edges of the vibration platform (Figure 3). The vibration procedure involved completing two rounds of 5-min vibrations followed by a 2-min break after each round.

### 2.5. Cognitive assessment: modified Stroop test

In this study, the modified Stroop test was programmed and administered using PsychoPy v2023.2 [41] to assess reaction times in young adults. The modified Stroop test is a measure of high reliability and validation. We selected the Stroop task as a validated measure of executive function, specifically targeting selective attention and inhibition, which are particularly sensitive to transient changes in arousal and autonomic modulation [31]. The tests were stable over long periods and easy to administer and score [36]. The decision to focus exclusively on executive function was theoretically grounded in evidence that this domain is particularly responsive to acute physiological and arousal-based modulation. Executive processes, such as selective attention and inhibition, are supported by prefrontal networks that rapidly adapt to transient somatosensory stimulation and autonomic fluctuations. Studies of acute exercise consistently report selective improvements in executive control rather than in memory or visuospatial domains, likely due to the prefrontal cortex's sensitivity to catecholaminergic and arousal-related influences [29],[33].

**Figure 4.**
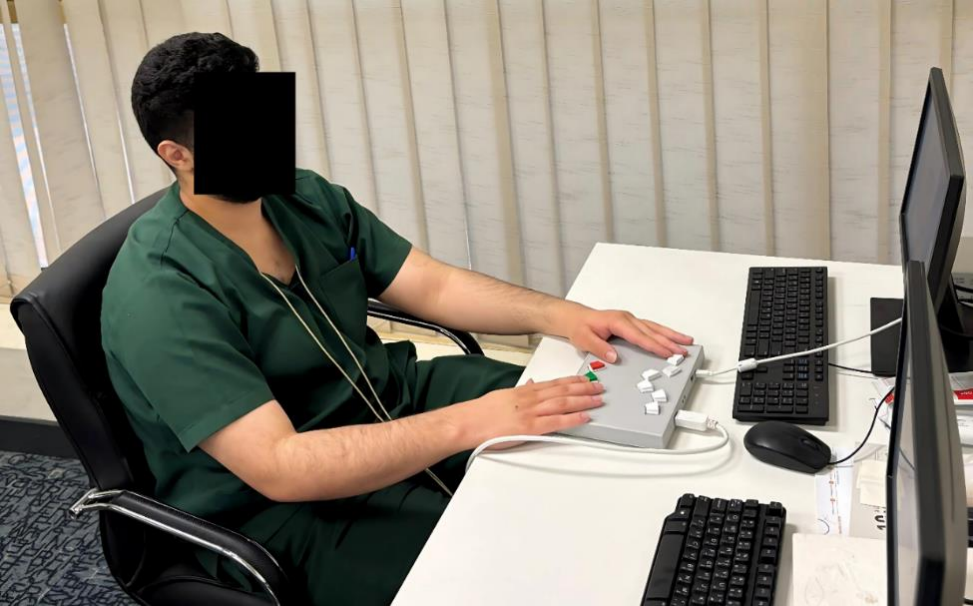
Modified Stroop test settings.

Because WBVT produces short-lived somatosensory stimulation and mild arousal without sustained metabolic demand, executive function provides the most sensitive and theoretically justified target for detecting immediate cognitive modulation following a single WBVT session. The test involved presenting 300 **color-word stimuli** (red, green, and orange) on a 15-inch LCD monitor (1920 × 1080 resolution), positioned at a 60 cm viewing distance. Each trial consisted of a 500 ms fixation cross followed by the stimulus (displayed until response or a maximum of 2000 ms), with an interstimulus interval of 1000 ms. The test began 2 min after cessation of WBVT to minimize confounding effects of postural instability and to capture immediate post-intervention effects. The stimuli were displayed under two conditions: **congruent**, where the word's meaning matched its font color (e.g., the word “red” displayed in red), and **incongruent**, where the word's meaning and font color conflicted (e.g., the word “red” displayed in green). The **incongruent condition** of the modified Stroop test was used to assess the effects of WBVT on **attention and inhibitory control.** Participants were told to press a button to indicate their response, choosing “Yes” when the color name aligned with the color of its presentation and “No” when it did not (See Figure 4). Stimuli were presented in a randomized sequence to minimize order effects. The main focus was on RT, as the metric was examined under three scenarios: (i) the overall response time (RT, for all stimuli combined), (ii) RT for congruent stimuli, and (iii) RT for incongruent stimuli.

This task setup allows for evaluating core components of the Stroop paradigm, including interference effects, selective attention, and response inhibition. The modifications in response handling, using a yes/no choice-based classification system, along with the selection of stimuli in the study, provide a customized approach to assess cognitive processing speed and executive function in younger individuals. Specific performance metrics based on RT and accuracy were used to deduce the following parameters:

i. Stroop effect or cognitive interference, which is a phenomenon that involves the interference of conflicting information in the brain's processing system. The Stroop effect measures the delay in response times when comparing incongruent and congruent conditions due to the effort needed to overcome conflict [32]. Resolving conflict becomes more challenging when there is an interference effect.

Stroop Interference effect=mean RT (Incongruent)−Mean RT (Congruent) (1)

ii. Selective attention represents the ability to focus on relevant information while ignoring distractions [42]. It can be assessed by accuracy rates and RT in the congruent condition:

$$\text{Accuracy in Congruent Trials }\left(\text{\%}\right)=\frac{\text{Correct Responses }(\text{Congruent})}{\text{Total Congruent Trials}}\times 100$$ (2)

Mean RT in congruent trials is also considered. Faster RTs in this condition suggest better attentional control and more efficient cognitive processing.

iii. Response inhibition (cognitive control). This refers to the ability to suppress automatic responses to conflicting stimuli [43] and can be assessed using accuracy and reaction time in incongruent trials:

$$\text{Accuracy in Congruent Trials }\left(\text{\%}\right)=\frac{\text{Correct Responses }(\text{Inongruent})}{\text{Total Inongruent Trials}}\times 100$$ (3)

$$\text{Incongruent RT vs}.\text{ Congruent RT Ratio}=\frac{\text{Mean RT }(\text{Congruent})}{\text{Mean RT }(\text{Incongruent})}$$ (4)

A higher ratio indicates a greater cognitive load required to override automatic responses.

**Figure 5.**
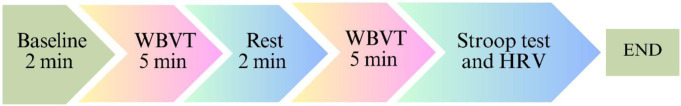
Schematic representation of the whole-body vibration therapy (WBVT) session protocol. The session began with a 2-min baseline period, followed by a 2-min rest. Participants then underwent two 5-min bouts of WBVT, each separated by a 2-min rest period. The session concluded with the administration of the modified Stroop test and heart rate variability (HRV) measurement.

The protocol for each measurement in standing posture for WBVT and the Stroop test is shown in Figure 5.

### 2.6. HRV recording and analysis

Continuous electrocardiography (ECG) was recorded using a PowerLab interface with LabChart software (ADInstruments, Dunedin, New Zealand) at a sampling frequency of 1000 Hz. Electrodes were placed in a standard Lead II configuration after skin preparation. Participants rested quietly for 5 min before the Stroop task, during which baseline HRV was recorded. ECG acquisition continued during Stroop testing and post-WBVT testing to capture HRV under three conditions: (i) baseline at rest, (ii) during the pre-WBVT Stroop task, and (iii) during the post-WBVT Stroop task. R–R intervals were artifact-corrected using a 5% threshold filter and visually inspected for ectopic beats, which were interpolated using a cubic spline method.

Frequency-domain parameters (LF, HF, LF/HF ratio, total power) were selected in line with The Task Force of the European Society of Cardiology and the North American Society of Pacing and Electrophysiology (1996) recommendation [44], as they provide robust indices of sympathetic–parasympathetic balance relevant for acute interventions. The following indices were analyzed: (i) low-frequency power (LF, 0.04–0.15 Hz): mixed sympathetic and parasympathetic influence; (ii) high-frequency power (HF, 0.15–0.40 Hz): vagally mediated parasympathetic activity; (iii) LF/HF ratio: sympathovagal balance, higher values indicate a shift toward dominance or a decrease in vagal tone; and (iv) total power: overall autonomic variability. Finally, a stress index (calculated as Baevsky's Stress Index) was also computed as a measure of autonomic activation.

Nonlinear measures (e.g., detrended fluctuation analysis) were not included due to the short recording epochs (<5 min), which limit reliability for these indices. Future work should incorporate both time- and nonlinear domain measures. Additionally, neither participants nor assessors were blinded to the intervention, given the obvious perceptual differences between vibration and standing conditions. However, outcome analyses (reaction time and HRV metrics) were performed automatically using software to minimize observer bias.

### 2.7. Statistical data analysis

All data were analyzed using the Statistical Package for Social Sciences (SPSS) (Version 26, IBM Corp., Armonk, NY). Descriptive statistics (mean ± standard deviation, SD) were computed for all dependent variables. To examine changes across the three experimental conditions (baseline, Stroop, and post-vibration), a one-way repeated measures ANOVA was conducted for physiological measures, including the stress index, heart rate, and HRV parameters (LF, HF, LF/HF ratio, and total power). Effect sizes were interpreted using Cohen's conventions: η² = 0.01 (small), η² = 0.06 (medium), η² ≥ 0.14 (large). Partial eta squared (η²) values are reported. For cognitive measures, paired-sample t-tests were used to compare pre- and post-vibration conditions for reaction times (total, congruent, incongruent), Stroop interference scores, accuracy percentages, and error counts. The Stroop interference effect was calculated as the difference between incongruent and congruent mean reaction times. Additional metrics for selective attention and response inhibition were evaluated using mean accuracy and response time ratios. The significance level of p < 0.05 was considered statistically significant.

## 3. Results

### 3.1. Participant characteristics

All 36 participants (18–25 years; 18 females, 18 males) completed both testing sessions. No adverse events, dropouts, or protocol deviations were recorded. Baseline demographic characteristics are presented in Table 2. All participants tolerated the WBVT protocol without discomfort or dizziness.

**Table 2.** Baseline characteristics of the sample of healthy young adults (n = 36).

**Characteristic**	**Value**
**General**	
Sex (male/female)	18/18
Age (years)	20.57 ± 1.84
**Anthropometry**	
Height (cm)	180.80 ± 7.18
Body mass (kg)	84.60 ± 11.36
Body mass index (kg/m²)	26.11 ± 9.27
**Health status**	
Medical history (self-reported)	None (100%)
Previous WBVT experience	None (100%)
Caffeine intake prior to sessions	None (100%)
**Physical activity (self-reported)**	
Vigorous activity per week (min)	50.55 ± 15.07
Moderate activity per week (min)	38.26 ± 14.59

Note: Values are presented as mean ± standard deviation (SD) unless otherwise indicated. WBVT, whole-body vibration therapy.

### 3.2. Autonomic function measures

A one-way repeated measures ANOVA was conducted to examine changes in physiological parameters across three conditions: baseline, pre-vibration Stroop test, and post-WBVT Stroop test. A marginally significant increase in the stress index was observed during the Stroop condition compared to baseline (Wilk's Lambda = 0.84, F [2,34] = 3.236, *p* = 0.052, η² = 0.16), suggesting a moderate effect size. Post-hoc analysis revealed a statistically significant increase in the stress index from baseline to the pre-vibration Stroop condition (*p* = 0.043). However, no significant differences were found between the pre-vibration Stroop and post-vibration Stroop conditions (p = 0.972) or between baseline and post-vibration Stroop (p > 0.99).

The heart rate remained stable across all conditions, with means ranging between 80.38 and 81.66 bpm. The LF/HF ratio showed a slight reduction post-vibration (from 3.15 to 2.71), suggesting a trend toward parasympathetic dominance, although not statistically significant. Total power, an indicator of overall autonomic activity, declined from 3006.11 ± 1745.53 to 2053.35 ± 1479.40, indicating reduced physiological arousal, potentially due to adaptation or relaxation post-exercise. These outcomes are summarized in Table 3. WBVT may modulate autonomic function by lowering the stress index and promoting parasympathetic recovery post-task, though these effects were subtle and not statistically significant.

### 3.3. Stroop Task performance

**Figure 6.**
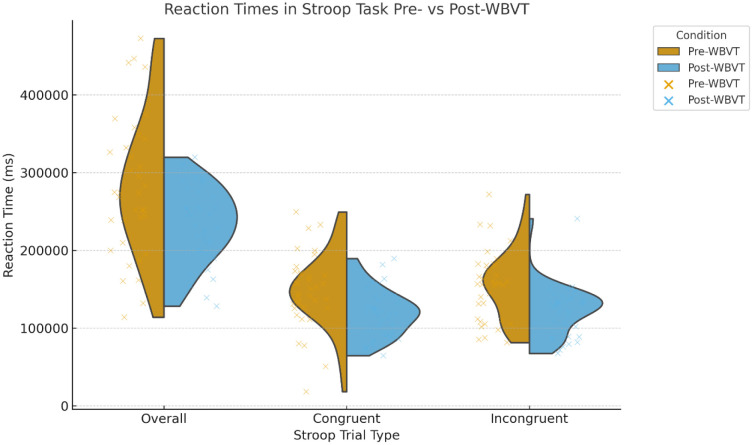
Reaction times (RT) in the modified Stroop task pre- and post-whole-body vibration therapy (WBVT). RainCloud plots display the distribution (violin), individual participant values (dots), and group comparison for overall, congruent, and incongruent conditions. Overall RT represents the mean reaction time calculated across all Stroop stimuli (both congruent and incongruent trials combined), providing a composite measure of general processing speed. WBVT was associated with significantly faster reaction times across all conditions, while accuracy remained stable.

RTs derived from the modified Stroop test showed robust improvements following WBVT. Mean overall RT decreased significantly from pre- to post-WBVT (296,304 ± 95,300 ms vs. 235,573 ± 54,766 ms, p < 0.001). Both congruent (152,639 ± 51,318 ms vs. 119,228 ± 28,534 ms, p < 0.001) and incongruent trials (143,664 ± 47,195 ms vs. 116,345 ± 32,311 ms, p < 0.001) demonstrated significant reductions in response latency (Table 4 and Figure 6). For clarity, “overall reaction time” refers to the average latency across all trials (both congruent and incongruent), representing a composite index of general cognitive processing speed.

The Stroop interference score (incongruent – congruent RT) did not significantly change (94.8 ± 156.4 ms vs. 100.5 ± 89.8 ms, p = 0.836), suggesting that while overall processing speed improved, conflict resolution ability remained stable. Accuracy remained high and unaffected by WBVT. In congruent trials, accuracy was 88.9% ± 3.1% pre- and 88.7% ± 4.1% post-WBVT (p = 0.785). In incongruent trials, accuracy was 97.4% ± 2.3% pre- and 97.9% ± 2.0% post-WBVT (p = 0.278). The incongruent/congruent RT ratio slightly decreased from 0.91 ± 0.12 to 0.89 ± 0.07 (p = 0.170), indicating preserved inhibitory control. Error rates were negligible and unchanged. Together, these findings indicate that WBVT enhanced cognitive processing speed without compromising accuracy or executive control.

**Table 3.** Descriptive statistics and repeated measures analysis of variance (ANOVA) results for stress index and heart rate variability (HRV) parameters across experimental conditions.

**Variable**	**Baseline** **(mean ± SD)**	**Pre-vibration Stroop** **(mean ± SD)**	**Post-vibration Stroop** **(mean ± SD)**	**F**	***p*-value**	**n^2^** (effect size)
Stress Index	9.85 ± 4.24	11.09 ± 4.28	10.42 ± 3.29	3.236	0.052	0.160
Mean heart rate	81.66 ± 12.69	80.38 ± 8.29	80.42 ± 6.84	0.342	0.713	0.020
LF (n.u.)	68.90 ± 16.08	68.24 ± 13.97	64.75 ± 14.36	1.777	0.184	0.095
HF (n.u.)	31.04 ± 16.04	31.02 ± 13.38	35.17 ± 14.35	2.120	0.136	0.111
Total power	3006.11 ± 1745.53	2332.78 ± 1137.58	2053.35 ± 1479.40	1.241	0.302	0.068
LF/HF ratio	2.99 ± 1.80	3.15 ± 2.12	2.71 ± 2.17	1.208	0.311	0.066

Note: HF, high frequency; LF, low frequency; SD, standard deviation. p = significant difference (2-tailed) at 0.05 level.

**Table 4.** Stroop task outcomes pre- and post-WBVT (n = 36).

**Variable**	**Pre-WBVT (mean ± SD)**	**Post-WBVT (mean ± SD)**	**Mean difference**	***t*-value**	***p*-value**
**Overall reaction time (ms)**	296,304 ± 95,300	235,573 ± 54,766	60,731	4.40	<0.001
**Congruent RT (ms)**	152,639 ± 51,318	119,228 ± 28,534	33,411	3.85	<0.001
**Incongruent RT (ms)**	143,664 ± 47,195	116,345 ± 32,311	27,320	4.12	<0.001
**Stroop interference (ms)**	94.8 ± 156.4	100.5 ± 89.8	−5.7	−0.21	0.836
**Accuracy, congruent trials (%)**	88.9 ± 3.1	88.7 ± 4.1	0.2	0.28	0.785
**Accuracy, incongruent trials (%)**	97.4 ± 2.3	97.9 ± 2.0	−0.5	−1.10	0.278
**Incongruent/congruent RT ratio**	0.91 ± 0.12	0.89 ± 0.07	0.03	1.40	0.170

Note: RT, reaction time; SD, standard deviation; WBVT, whole body vibration therapy. p = significant difference (2-tailed) at 0.05 level.

### 3.4. Exploratory within-subject correlation analysis

To explore potential covariation between cognitive and autonomic responses, within-subject correlation analyses were conducted between change scores in Stroop reaction time (ΔRT) and autonomic indices (ΔStress Index, ΔLF, and ΔHF). Change scores were calculated as post-WBVT minus pre-WBVT values. Repeated-measures correlation analysis (rmcorr) [45] was used to account for non-independence across repeated measures. As shown in Table 5, no significant within-subject associations were observed between ΔRT and any autonomic parameter (all p > 0.10). Although directionally negative correlations were noted between ΔRT and ΔStress Index (r = −0.21) and between ΔRT and ΔLF (r = −0.18), these trends did not reach statistical significance. These results indicate that individual differences in cognitive improvement following WBVT were not systematically related to autonomic modulation.

**Table 5.** Exploratory within-subject correlation between change in Stroop reaction time (ΔRT) and autonomic indices (n = 36).

**Parameter (Δ)**	**r_rm**	**95% CI**	***p*-value**	**Interpretation**
**Stress Index**	−0.21	−0.45 to 0.09	0.181	Weak, non-significant negative correlation
**LF (n.u.)**	−0.18	−0.43 to 0.11	0.243	Weak, non-significant negative correlation
**HF (n.u.)**	0.09	−0.20 to 0.36	0.567	No significant association

Note: r_rm = repeated-measures correlation coefficient. Δ indicates post-WBVT minus pre-WBVT values. No associations reached statistical significance (all p > 0.10).

## 4. Discussion

This study examined the acute effects of WBVT on cognitive performance and ANS function in healthy young adults. The findings suggest that WBVT is associated with improved RT on inhibitory control and selective attention tasks (i.e., core components of prefrontal cognitive regulation that are sensitive to transient physiological and autonomic changes) without compromising accuracy or producing measurable autonomic imbalance [33]. In this study, the term *selective attention task* refers specifically to the modified Stroop test administered here; all reported reaction time and accuracy metrics are derived from that single Stroop paradigm. Together, these results contribute to an emerging body of evidence supporting WBVT as a potentially useful, non-pharmacological approach to enhancing cognitive efficiency (i.e., faster information processing or reduced RT achieved without a loss of task accuracy), for example, an increase in processing speed that does not trade off with performance accuracy or error rate [16],[46]. In our data, this is operationalized as decreased mean RT for overall, congruent, and incongruent trials on the Stroop test while accuracy remained stable. At the same time, the interpretation of these findings requires caution, as several aspects of the results remain exploratory and warrant replication in larger, rigorously controlled studies.

Moreover, the findings from this study should be considered in the context of the literature on both active and passive exercise. Active aerobic and resistance exercise reliably alters HRV and improves several cognitive domains, with mechanisms attributed to increased cerebral perfusion, neurotrophic signaling, and autonomic adaptations. Passive exercise modalities (including WBVT) may share some of these pathways, for example, by providing peripheral somatosensory input that transiently increases cortical excitability and arousal, but likely differ in magnitude and temporal dynamics because of the absence of large-scale central motor activation [46],[47]. Reconciling discrepancies across studies requires careful attention to intervention dose (frequency, amplitude, duration), mode (vertical vs. side-alternating), population, and timing of cognitive assessment (immediate vs. delayed).

### 4.1. Cognitive outcomes

RT improvements across congruent, incongruent, and total Stroop task conditions indicate that WBVT may facilitate faster information processing and attentional deployment. These results align with prior studies suggesting that vibration-based somatosensory stimulation can transiently increase cortical excitability in motor and prefrontal regions, facilitating executive processes such as attentional control and response selection [20],[21],[48]. The study by Regterschot et al. [16] is highly relevant: they also used a Stroop-type selective attention paradigm following WBVT and reported improvements in attentional control. Our findings similarly show faster Stroop RT post-WBVT, although we extend the literature by including HRV measures. Notably, while Regterschot et al. [16] reported improvements in inhibitory control following WBVT, our findings primarily indicate faster response latencies without concomitant changes in accuracy or Stroop interference. This pattern suggests an enhancement in processing speed and attentional deployment rather than a measurable alteration in conflict-driven inhibitory control in this young, high-performing cohort.

This pattern is compatible with arousal-based models of performance, such as the Yerkes–Dodson law, which proposes that mild physiological arousal can optimize attentional resources and processing speed [13],[49]. In this context, WBVT may function as a mild, controlled arousal stimulus that enhances alertness without inducing sympathetic overactivation that can degrade performance accuracy. However, we caution against extrapolating these results beyond the protocol tested here. Because we evaluated a single bout (two 5-min bouts at ~27 Hz, separated by 2 min), we cannot determine how frequency, amplitude, or session duration modulate cognitive outcomes. Definitive dose–response inferences would require experimental comparisons across multiple WBVT parameter sets.

The absence of a significant change in Stroop interference scores is notable. Interference effects are a more specific marker of higher-order cognitive control, relying heavily on prefrontal networks for conflict monitoring and inhibition [50],[51]. The lack of improvement here may indicate that WBVT preferentially benefits lower-level processes, such as sustained attention and psychomotor speed, rather than more complex executive control functions. Alternatively, ceiling effects in a high-functioning, young adult cohort could have masked subtle gains in more demanding cognitive domains.

Previous reports vary in both exposure characteristics and target populations. For example, some studies reporting cognitive gains used acute exposures similar to ours (single-session protocols), whereas others implemented chronic interventions (multiple sessions per week over several weeks). Differences in vibration mode (side-alternating vs. vertical), frequency, amplitude, and population (older adults, clinical groups, healthy young adults) likely contribute to inconsistent findings [18],[52]. However, whether such effects are mediated by changes in neural excitability, sensorimotor integration, or attentional priming remains unresolved. Mechanistic studies incorporating neuroimaging, electrophysiological, or neurochemical markers will be essential to determine the underlying pathways.

### 4.2. Autonomic outcomes

Physiological data showed an expected transient increase in the stress index during cognitive challenge, consistent with sympathetic activation during attentional tasks [53]. Following WBVT, the stress index values approached baseline, suggesting but not confirming a potential trend toward autonomic recovery. Importantly, no statistically significant changes were detected in standard HRV parameters, including LF, HF, LF/HF ratio, or total power. Although the directional shifts in some HRV measures were consistent with prior reports of vibration-related modulation of vagal activity [54],[55], the lack of statistical significance requires that these trends be interpreted cautiously.

Potential confounders that can influence HRV and cognitive measures include respiration rate and depth, recent sleep quality, caffeine intake, posture changes, and emotional state, all of which can modulate autonomic tone and cortical arousal [56]–[59]. We sought to minimize some of these: participants were instructed to refrain from vigorous exercise, caffeine, and smoking for at least two hours before testing, and sessions were scheduled at similar daytime hours. However, respiration was not paced or continuously monitored during HRV recording, and we did not objectively verify sleep or stress levels before each visit. These omissions increase physiological variance and may reduce sensitivity to detect small autonomic effects; consequently, the null HRV findings should be interpreted cautiously [60]. Future studies should include respiratory monitoring, standardized pre-test sleep/stress diaries, and/or paced breathing during recordings to reduce these sources of variability.

### 4.3. Integrated interpretation

The concurrent improvement in RT and normalization of stress indices (albeit non-significant) raises the possibility that WBVT could promote more efficient psychophysiological integration, that is, the coordinated regulation of cognitive and autonomic systems during challenge. This hypothesis is consistent with models describing bidirectional communication between prefrontal, limbic, and vagal pathways in supporting adaptive performance under stress [61]. However, the present study cannot determine whether cognitive gains are mediated by autonomic modulation, whether autonomic recovery is secondary to cognitive improvements, or whether both are parallel responses to a common arousal-related mechanism. Experimental mediation analyses and longitudinal studies are needed to clarify these relationships.

Alternative explanations should also be considered. The observed benefits might reflect nonspecific effects, such as posture changes, increased proprioceptive input, or expectancy/placebo influences rather than vibration-specific mechanisms [62]. A sham-controlled design, ideally with blinding, will be critical in future work to disentangle true mechanistic effects from general arousal or attentional priming.

To further explore potential brain–heart interactions, an exploratory within-subject correlation analysis was conducted between individual changes in Stroop reaction time and autonomic indices (stress index, LF, HF). No significant correlations were found (all p > 0.10), suggesting that improvements in cognitive speed were not directly coupled with autonomic adjustments in this cohort.

In summary, a single session of WBVT was associated with faster Stroop reaction times in our sample, but the lack of a sham control and the absence of robust autonomic changes prevent strong causal inferences. Future sham-controlled, parameterized (dose–response) studies incorporating respiratory control and within-subject neurophysiological measures are required to determine whether WBVT directly modulates brain–heart coupling or whether observed cognitive benefits reflect practice, time, or generalized arousal effects.

### 4.4. Limitations and future directions

Several limitations should be acknowledged. First, the study used a homogeneous sample of healthy, young university students, limiting generalizability to older adults or clinical populations who may respond differently to WBVT. Second, although a 7-day interval was used to reduce practice effects, repeated Stroop testing may still have introduced learning or familiarity effects; using alternate forms of the test could reduce this risk. Third, no sham or blinding procedure was implemented, leaving open the possibility of placebo effects. In addition to placebo and expectancy effects, simple “time effects” (e.g., practice, arousal changes across the session, or fatigue dissipation) can influence cognitive outcomes. Because we did not include a sham standing-control session with matched timing and experimental handling, we cannot definitively separate vibration-specific effects from time/practice effects. The within-subject counterbalancing partially mitigates but does not remove this concern; future protocols should include a sham vibration or standing control condition and/or multiple baseline sessions to quantify practice-related gains.

Fourth, respiration was not controlled during HRV recordings, introducing a known source of variability. Fifth, the study's sample size, while adequate for detecting moderate behavioral effects, may have been underpowered for detecting subtle autonomic changes. Sixth, the absence of pre-registration and the exploratory nature of the autonomic analyses mean that these findings should be considered hypothesis-generating rather than confirmatory. Finally, although exploratory within-subject correlation analyses were performed, the use of only two repeated measures per participant limits statistical power to detect within-subject associations [62]. Future studies with more repeated time points are recommended to robustly model brain–heart coupling dynamics.

Future studies should address these limitations by including sham-controlled and blinded conditions, larger and more diverse samples, broader cognitive test batteries, and mechanistic measures such as electroencephalography (EEG), functional near-infrared spectroscopy (fNIRS), or BDNF assays. Dose–response investigations examining the effects of vibration frequency, amplitude, and session duration would also help optimize protocols. Comparative trials contrasting WBVT with other interventions (e.g., aerobic exercise, mindfulness, or HRV biofeedback) will clarify its relative efficacy and inform clinical translation.

## 5. Conclusions

This study indicates that Stroop RT was faster after a single WBVT session, though practice or time effects cannot be ruled out in the absence of a sham control. Autonomic measures showed a non-significant trend toward autonomic normalization (i.e., elevated stress during cognitive challenge, tended to return toward baseline) after WBVT. This pattern suggests a recovery toward pre-task autonomic balance rather than sustained sympathetic activation. Overall, WBVT may transiently enhance processing speed without compromising accuracy or inducing autonomic imbalance, but confirmation in larger, sham-controlled studies is needed.

## Use of AI tools declaration

The authors acknowledge that ChatGPT 4.0, an AI language model developed by OpenAI, was used to assist in refining certain limited sections of this manuscript, specifically for language editing and improvement. All AI-assisted content was carefully reviewed and thoroughly edited by the authors to ensure accuracy, scientific rigor, and compliance with academic writing standards. The authors assume full responsibility for the content of this manuscript, including any parts enhanced with AI tools, and remain accountable for any breach of publication ethics.
